# Preliminary Study of Conformation and Drug Release Mechanism of Doxorubicin-Conjugated Glycol Chitosan, via *cis*-Aconityl Linkage, by Molecular Modeling

**DOI:** 10.3390/ijms12031672

**Published:** 2011-03-04

**Authors:** Thongchai Srinophakun, Jirapat Boonmee

**Affiliations:** Center of Excellence for Petroleum, Petrochemicals, and Advanced Materials, and Center for Advanced Studies in Industrial Technology, Department of Chemical Engineering, Kasetsart University, Bangkok 10900, Thailand; E-Mail: melody_tanna@hotmail.com

**Keywords:** drug release, molecular modeling, glycol chitosan, doxorubicin

## Abstract

An investigation of the structure and drug release mechanism of a drug delivery system is proposed on the basis of semi-empirical and *ab initio* computations in vacuum stage. *Cis*-aconityl linkage is used to improve the interaction between an anti-cancer agent, doxorubicin, and a glycol chitosan biopolymer. It has been found that the doxorubicin-conjugated glycol chitosan carrier has more stability. The stability is increased when the lengths of the polyethylene glycol (PEG) chains in the glycol chitosan biopolymer are increased. *Cis*-aconityl can release doxorubicin under appropriate environmental conditions. Relative energies of this mechanism in an acid condition, as determined by B3LYP/6-31G//PM3, are 122.41, 119.27, 160.18 and 222.22 kcal/mol, and by the B3LYP/6-31G//HF/6-31G method are 54.23, 109.28, 219.98 and 980.49 kcal/mol, with mono-, di-, tri-, and quanta-ethylene glycol, respectively. In a normal condition, the relative energies are above 300 kcal/mol for all reactions. Therefore, *cis*-aconityl will release doxorubicin in an acid solution but not in a normal condition. The glycol chitosan polymer can be degraded in an acid solution as well. Long PEG chains influence the release mechanism of doxorubicin. The proposed length of the PEG chain is di-ethylene glycol. These simulation results agree well with various reported experimental data.

## 1. Introduction

During the past decade there has been a growing interest in the investigation of polymeric carriers for their potential use in anticancer drug delivery. The development of chemotherapeutic agents for cancer treatment has resulted in overcoming some tumors. However, these new chemotherapeutic agents still have serious side effects, such as non-specific toxicity to normal tissues, which limits the dosage and uses of the drug [1]. Consequently, to decrease the toxicity of anticancer agents to normal tissues, the improvement of a drug delivery system for anti-cancer drugs is of interest [2].

Chitosan, a biopolymer which is obtained from chitin, is accepted as a biodegradable and nontoxic polymer. It has been widely used in the pharmaceutical and cosmetic industries as an antimicrobial compound. It is a cationic polysaccharide consisting primarily of repeating units of β-(1,4)-2-amino-deoxy-d-glucose (d-glucosamine) [3]. The applications of chitosan are limited because of its insolubility above pH 6. To increase the solubility of chitosan in water, one or two hydrogen atoms are removed from the amino groups of chitosan and some hydrophilic segments are introduced. Polyethylene glycol (PEG), as a hydrophilic segment of polymeric carriers [4], can freely dissolve over a wide pH range. For most anticancer drug carriers, the interactions between the anticancer drug and the core domain of the polymeric drug carrier rely on the balance of relatively weak interactions, such as hydrophobic, H-bonding, and electrostatic interactions [5,6].

Hence, polymeric carriers have a limitation because they disintegrate after dilution by body fluids, resulting in leakage of the anticancer drug into the blood vessels [7,8]. One strategy to develop a drug delivery system is conjugation of the anticancer drug with polymer chains by chemical linkage. This strategy can control the drug release rate and reduce the burst release [9,10].

Although various experimental measurements can provide information necessary for improving controlled drug delivery systems, it would be highly beneficial if one could predict their performance by using computational simulations. Consequently, we investigated the drug delivery system of doxorubicin-conjugated glycol chitosan via *cis*-aconityl linkage (GC-DOX) by a molecular modeling technique with two computed processes: semi-empirical PM3 and *ab initio* HF methods. Our hope is that these computational investigations can be used as a predictive tool for guiding and improving drug delivery system complexes. A better understanding of electronic structure can also help improve these drug delivery molecules.

In this article, we propose geometrical parameters of doxorubicin-conjugated glycol chitosan via *cis*-aconityl linkage (GC-DOX) and doxorubicin in glycol chitosan by H-bonding (GC/DOX), in order to compare the stability of these two carriers. The doxorubicin release mechanism is studied for solution effects, including the effect of the length of PEG chains.

## 2. Model and Methods

**Figure 1.**
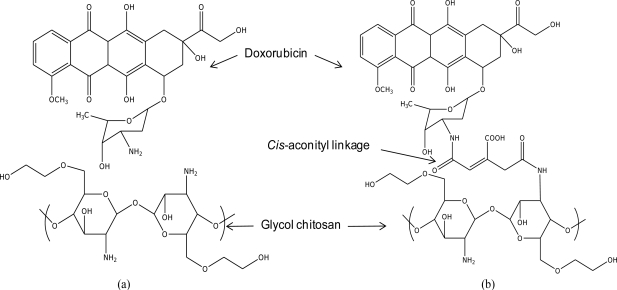
Chemical structures of (**a**) GC/DOX and (**b**) GC-DOX carriers.

The focused drug carrier model in this study is a doxorubicin-conjugated glycol chitosan polymer via *cis*-aconityl linkage (GC-DOX), as was used in Son *et al*. [1]. In order to confirm the improvement of the interaction between the drug and the polymer chain by *cis*-aconityl linkage, a GC/DOX carrier (see Figure 1) was modeled for comparison. Note: we used one molecule of doxorubicin conjugated with a tri-glucosamine ethylene glycol chain. The length of the PEG chain was increased from one up to five molecules in each drug carrier.

**Figure 2.**
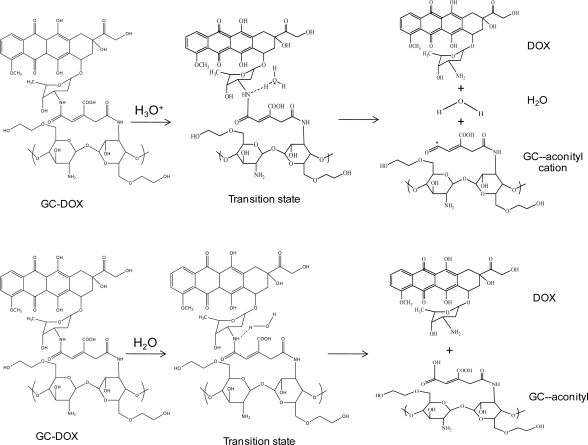
The proposed doxorubicin release mechanism in acid and normal conditions.

**Figure 3.**
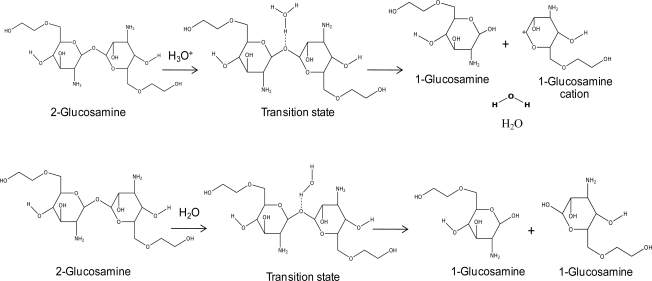
The proposed glycol chitosan degradation mechanism in acid and normal conditions.

In order to investigate doxorubicin release, a hydronium ion and water were used as models of acid and normal conditions, respectively. The geometries of these complexes of the doxorubicin release mechanism are shown in Figure 2. Glycol chitosan degradation in solution was also studied, as well as the mechanism of doxorubicin release from the *cis*-aconityl linkage, as shown in Figure 3.

Optimizations of model structure geometry were performed at the PM3 and HF/6-31G levels. PM3 is a suitable method when there is sufficient experimental data, while HF/6-31G needs only molecular structure information. We chose the density functional theory (DFT) with the B3LYP/6-31G functional, which was found to be appropriate for predicting electron structure, in order to calculate molecular energy. All calculations were implemented using the Gaussian 03W program [11] under the assumption of vacuum phase.

## 3. Results and Discussion

In this section, we report the optimized structures of the GC/DOX and GC-DOX carriers with various lengths of PEG chains, and the important structural parameters of these two carriers. Geometry optimizations of the reactant, transition state, and products of both the doxorubicin-releasing reaction from the linkage and the glycol chitosan-degradation reaction are represented by relative energies.

### 3.1. Optimal Structures and Molecular Energies

**Figure 4.**
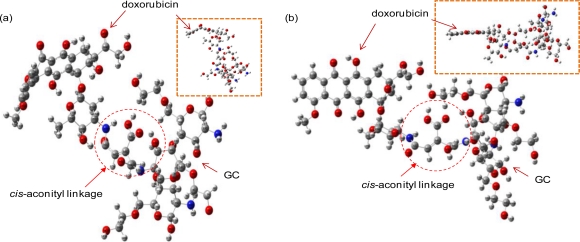
Optimal structures of the GC-DOX carrier by (**a**) PM3 and (**b**) HF/6-31G methods.

**Figure 5.**
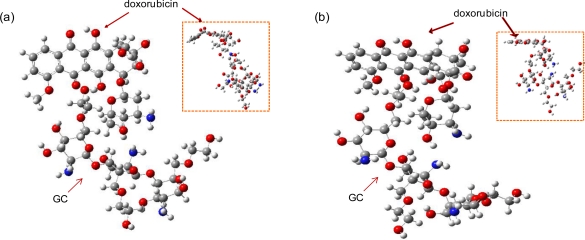
Optimal structures of the GC/DOX carrier by (**a**) PM3 and (**b**) HF/6-31G methods.

The optimal structures of the GC-DOX and GC/DOX carriers were obtained by both PM3 and HF/6-31G methods, as shown in Figures 4 and 5. It can be seen that doxorubicin in both GC-DOX and GC/DOX carriers, which is optimized by the HF/6-31G method, has the correct planar fused-ring structure, while the PM3 method results in a bent shape. Therefore, the HF/6-31G method can optimize the fused-ring structure better than the PM3 method. The HF/6-31G method can also better optimize GC-DOX carriers with long PEG chains. Optimized long PEG chains from HF/6-31G are straight, but they are distorted by the PM3 method.

The molecular energies of both GC/DOX and GC-DOX carriers are presented in Table 1. As is clearly shown, the molecular energies of the GC-DOX structure are generally lower than those of GC/DOX. In other words, GC-DOX carriers are more stable because the *cis*-aconityl can formulate the bond between the drug and the polymer. The N-H bond length between doxorubicin and *cis*-aconityl is 1.47 Å; this is shorter than the hydrogen bond, which has an approximate length of 1.8–2.0 Å. The N-H bond is a shorter bond because more electrons participate in bond formation. It is also a stronger bond; thus the GC-DOX carrier has more stability. The stability of both GC/DOX and GC-DOX molecules is increased according to the length of the ethylene glycol chains. For the computed stable geometry of GC/DOX with a mono-ethylene glycol molecule by HF/6-31G, the hydrogen bond distance between O-H groups of doxorubicin and the glycol chitosan polymer is 1.95 Å. For GC/DOX with penta-ethylene glycol molecules, the hydrogen bond is 1.86 Å. The interaction between doxorubicin and the glycol chitosan chain is compressed by increasing the ethylene glycol units in PEG chains.

### 3.2. Doxorubicin Release Mechanism

This simulation studied the effect of a solution on bond-breaking between doxorubicin and the *cis*-aconityl linkage. A hydronium ion (H_3_O^+^) was used as a model of an acid condition, and water (H_2_O) was used as a model of a normal condition. An estimation of the reaction can be calculated from the relative energy between the total molecular energy of the product and the total molecular energy of the reactant. The possibility of reaction is explained by the relative energy of each reaction step. If the relative energy of the product is negative, the reaction has a possibility of occurring because the product is stable [11].

#### 3.2.1. Acid Condition

**Figure 6.**
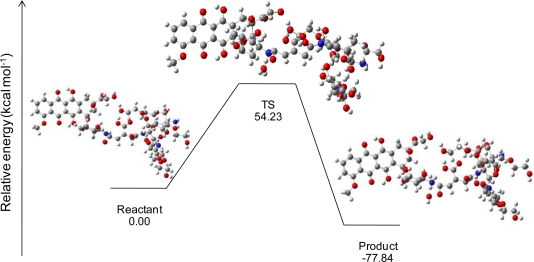
Relative energies and geometries of the stationary states of the doxorubicin release mechanism of GC-DOX with mono-ethylene glycol in an acid condition, by the HF/6-31G method.

In an acid condition, a hydronium ion reacts with the N-H bonding between doxorubicin and the *cis*-aconityl linkage. Finally, the complex in the transition state (TS) separates into three molecules: doxorubicin, glycol chitosan bonding with the *cis*-aconityl cation, and water. Geometry optimizations of doxorubicin released from GC-DOX with a mono-ethylene glycol complex by the HF/6-31G method are shown in Figure 6 with their relative energies. The relative energy in the transition state is greater than the reactant because the hydronium ion needs “activated energy” at least 54.23 kcal mol^−1^ to react with the doxorubicin and *cis*-aconityl bonding. Relative energies for the release mechanism of doxorubicin with a PEG chain are shown in Table 2.

The activated energies of doxorubicin released from each reaction in an acid condition by B3LYP/6-31G//PM3 are 122.41, 119.27, 160.18 and 222.22 kcal/mol with mono-, di-, tri- and quanta(ethylene glycol), respectively. On the other hand, B3LYP/6-31G//HF/6-31G gives energies of 54.23, 109.28, 219.98 and 980.49 kcal/mol for mono-, di-, tri-, and quanta(ethylene glycol), respectively. The length of the PEG chain influences the bond-breaking of doxorubicin and the linkage: the longer the PEG chains, the more difficult the bond-breaking. Since the PEG chains have a steric hindrance effect which disturbs the hydronium ion’s interaction with the nitrogen atom in doxorubicin, the reaction needs high activated energy.

#### 3.2.2. Normal Condition

In a normal condition, the water molecule is attracted to the N-H bonding between doxorubicin and the *cis*-aconityl linkage (see Figure 2). The activated energies of doxorubicin released from GC-DOX in a normal condition by the B3LYP/6-31G//PM3 method are 379.06, 342.03 and 433.77 kcal/mol with mono-, di- and tri(ethylene glycol), respectively. The respective energies from the B3LYP/6-31G//HF/6-31G method are 387.94, 325.67 and 444.78 kcal/mol, as shown in Table 3. According to both semi-empirical PM3 and *ab initio* HF/6-31G methods, the activated energies of the release mechanism of the three reactions in a normal condition are much higher than in an acid condition. This high amount of energy will obstruct the possibility of a reaction. Therefore, we stopped the simulation in normal condition at 3-PEG. Consequently, the *cis*-aconityl linkage cannot release the doxorubicin molecule from the GC-DOX carrier in a normal environment.

These results in acid and normal conditions correspond with the experimental data from Son *et al*. [1]. They studied doxorubicin released from DOX/GC-DOX in different pH media. Their report showed that the *cis*-aconityl linkage is pH-sensitive with hydrolysis [1,12] in an acidic environment. Doxorubicin was released continuously from nanoaggregations at pH 4, while the release of doxorubicin was almost negligible at pH 7. The bloodstream is a normal environment (pH 7.4), which has no effect on doxorubicin and linkage bonding. Since *cis*-aconityl is pH-sensitive and helps stabilize the formation, it can control drug leakage in blood vessels before the target cells are reached. When these drug carriers contact the target cell, they can release the drug via lysozymes from lysosome organelles which function well in an acid solution of pH 4.5.

### 3.3. Glycol Chitosan Degradation

During doxorubicin release, glycol chitosan polymers will also be degraded. This part of the simulation used di-glucosamine(ethylene glycol) to represent the glycol chitosan polymer. The reaction of glycol chitosan degradation was studied under both acid and normal conditions; doxorubicin release under these conditions was studied as well. The reaction possibility can be calculated from the relative energy between the total molecular energy of the product and the total energy of the reactant, in the same manner as described in the previous section.

#### 3.3.1. Acid Condition

**Figure 7.**
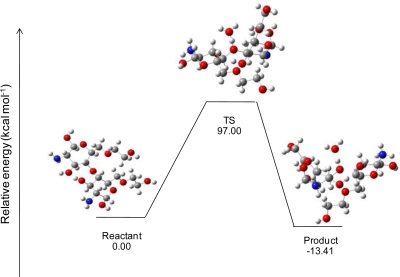
Relative energies and geometries of stationary states of the glycol chitosan bond-breaking mechanism in an acid condition, by the HF/6-31G method.

The results showed that the hydrogen atom in the hydronium ion interacts with the oxygen atom of di-glucosamine(ethylene glycol) in the transition state. There are three different molecules in the final step: the glucosamine(ethylene glycol) cation, glucosamine(ethylene glycol), and water. The reaction steps of glycol chitosan degradation in an acid condition by the HF/6-31G method are shown in Figure 7. The effects of the lengths of ethylene glycol chains on the reaction are shown in Table 4.

The activated energies of the reaction of di-glucosamine(ethylene glycol) in an acid condition are 145.45, 91.12, 61.41, 43.04 and 33.63 kcal/mol for mono-, di-, tri-, quanta- and penta(ethylene glycol), respectively, by the B3LYP/6-31G//PM3 method. The activated energies from B3LYP/6-31G//HF/6-31G are 97.00, 62.59, 44.11, 32.31 and 35.31 kcal/mol for mono-, di-, tri-, quanta- and penta(ethylene glycol), respectively. Polyethylene glycol (PEG) affects glycol chitosan bond-breaking. When the length of the ethylene glycol chain is increased, it can decrease the activated energy of the reaction.

In the product stage, the semi-empirical PM3 method shows that reactions which consist of mono-, di-, tri- and quanta (ethylene glycol) have the potential to occur. However, since the relative energy of the product in the di-glucosamine penta(ethylene glycol) bond-breaking reaction has a positive value, the product is unstable, and hence will not occur. Reactions that do not have potential when simulated by the *ab initio* HF/6-31G method are di-glucosamine with quanta- and penta(ethylene glycol).

#### 3.3.2. Normal Condition

For the glucosamine (ethylene glycol) bond-breaking mechanism in a normal condition, a water molecule was used as a model. The products are two glucosamine (ethylene glycol) molecules. The relative energies of their reactions with the effect of the PEG chain are presented in Table 5.

B3LYP/6-31G//PM3 simulation demonstrates that the activated energies in a normal condition are 456.92, 364.29, 412.88 and 409.54 kcal/mol for mono-, di-, tri- and quanta(ethylene glycol), respectively; while B3LYP/6-31G//HF/6-31G gives respective energies of 474.96, 460.33, 464.98 and 451.65 kcal/mol for mono-, di-, tri- and quanta(ethylene glycol). It can be seen that all these reactions consume high amounts of activated energy, and that the product energies of all reactions are positive values, which point to their unstable state. Therefore, glycol chitosan cannot be degraded in a normal solution. The simulation results in this section correspond well with experimental results from Saito *et al.* [13], who reported that chitosan could be stable in a water solution but is degraded in an acidic solution, because H^+^ in acid can more easily act on the GC-DOX bond than H_2_O can.

The pathway of GC-DOX carriers starts from injection into a blood vessel. The loaded doxorubicin in GC-DOX carriers cannot be released under the pH 7.4 condition of blood vessels, which corresponds with the simulation. After the GC-DOX carriers are distributed to cancer cells, the endocytosis mechanism will transport the GC-DOX carriers into vesicles. These vesicles permeate the endosome and reach the lysosome compartments. There, doxorubicin molecules are released by lysosomal enzymes called lysozymes. The released doxorubicin molecules move to the nucleus in order to inhibit the growth of cancer cells. Following the release of doxorubicin, some of the used drug carriers are degraded by lysozymes and are then reabsorbed by vesicles in a process called exocytosis, to later be completely digested in other compartments. Finally they are excreted from the body in urine via the kidneys or in bile [14].

## 4. Conclusions

In this article, we analyzed the stability of the drug carrier doxorubicin-conjugated glycol chitosan polymer via *cis*-aconityl linkage (GC-DOX), and the doxorubicin release mechanism by a molecular modeling method. The GC-DOX carrier improved the interaction by controlling the leakage of loaded doxorubicin from carrier molecules. The *cis*-aconityl linkage can release doxorubicin even under the acidic environment effect of lysozyme. In the human body, doxorubicin is released by lysozymes, which work well under acid conditions. After the doxorubicin is released, the glycol chitosan carrier will then be degraded by this enzyme. From the simulation, the PEG chain plays a major role in drug carrier formation and the drug release reaction. The optimal length of the PEG chain for this GC-DOX system is di-ethylene glycol (2-PEG), due to the fact that long PEG chains influence the doxorubicin release mechanism and the degradation of glycol chitosan. Even though there was satisfactory agreement with experimental data from the literature of the simulations by the semi-empirical PM3 and *ab initio* HF/6-31G methods under the assumption of vacuum phase, we would not recommend that this result be directly considered as it is still experimental work.

## Figures and Tables

**Table 1.** Molecular energies of GC/DOX and GC-DOX molecules with effect of ethylene glycol chain length.

**Length of ethylene glycol**	**Molecular energy (A.U.)**			
	**B3LYP/6-31G//PM3**		**B3LYP/6-31G//HF/6-31G**	

	**GC/DOX**	**GC-DOX**	**GC/DOX**	**GC-DOX**
Mono-ethylene glycol	−4237.555	−4767.910	−4237.734	−4768.159
Di-ethylene glycol	−4698.891	−5229.235	−4699.082	−5229.489
Tri-ethylene glycol	−5160.238	−5690.576	−5160.426	−5690.846
Quanta-ethylene glycol	−5621.580	−6151.916	−5621.771	−6152.184
Penta-ethylene glycol	−6082.922	−6613.259	−6083.115	−6613.527

**Table 2.** Relative energies of the doxorubicin release mechanism in an acid condition.

**Length of PEG**	**Reaction steps**	**Relative energy (kcal/mol)**	
		**B3LYP/6-31G//PM3**	**B3LYP/6-31G//HF/6-31G**
1-PEG	Reactant	0.00	0.00
	TS	122.41	54.23
	Product	−95.64	−77.84
2-PEG	Reactant	0.00	0.00
	TS	119.27	109.28
	Product	−113.31	−77.61
3-PEG	Reactant	0.00	0.00
	TS	160.18	219.98
	Product	−96.31	−69.53
4-PEG	Reactant	0.00	0.00
	TS	222.22	980.49
	Product	−99.65	−73.39

**Table 3.** Relative energies of the doxorubicin release mechanism in a normal condition.

**Length of PEG**	**Reaction steps**	**Relative energy (kcal/mol)**	
		**B3LYP/6-31G//PM3**	**B3LYP/6-31G//HF/6-31G**
1-PEG	Reactant	0.00	0.00
	TS	379.06	387.94
	Product	−93.70	−43.97
2-PEG	Reactant	0.00	0.00
	TS	342.03	325.67
	Product	−95.06	−58.54
3-PEG	Reactant	0.00	0.00
	TS	433.77	444.78
	Product	−107.88	−50.78

**Table 4.** Relative energies of the glycol chitosan bond-breaking mechanism in an acid condition.

**Length of PEG**	**Reaction steps**	**Relative energy (kcal/mol)**	
		**B3LYP/6-31G//PM3**	**B3LYP/6-31G//HF/6-31G**
1-PEG	Reactant	0.00	0.00
	TS	145.45	97.00
	Product	−17.77	−13.41
2-PEG	Reactant	0.00	0.00
	TS	91.12	62.59
	Product	−16.60	−18.70
3-PEG	Reactant	0.00	0.00
	TS	61.41	44.11
	Product	−6.61	−14.00
4-PEG	Reactant	0.00	0.00
	TS	43.04	32.68
	Product	−4.06	18.74
5-PEG	Reactant	0.00	0.00
	TS	33.63	35.31
	Product	62.85	27.52

**Table 5.** Relative energies of the glycol chitosan bond-breaking mechanism in a normal condition.

**Length of PEG**	**Reaction steps**	**Relative energy (kcal/mol)**	
		**B3LYP/6-31G//PM3**	**B3LYP/6-31G//HF/6-31G**
1-PEG	Reactant	0.00	0.00
	TS	456.92	474.96
	Product	22.75	3.87
2-PEG	Reactant	0.00	0.00
	TS	364.29	460.33
	Product	17.71	3.50
3-PEG	Reactant	0.00	0.00
	TS	412.88	464.98
	Product	14.70	2.69
4-PEG	Reactant	0.00	0.00
	TS	409.54	451.65
	Product	10.71	0.81
